# Anti-Alpha-Gal Antibodies Against Gangliosides: Preliminary Data on a New Autoimmune Target in Alzheimer’s Disease Patients

**DOI:** 10.3390/ijms27146190

**Published:** 2026-07-10

**Authors:** Filippo Naso, Alessandro Gandaglia, Giulio Sturaro, Alessio Lepore, Alessia Arcaro, Fabrizio Gentile, Alfonso Di Costanzo, Antonella Angiolillo

**Affiliations:** 1 Biocompatibility Innovation S.r.l., Via Lorenzo de Antoni, n.17/19, 35042 Este, Italy; f.naso@bci-biocompatibility.com (F.N.); a.gandaglia@bci-biocompatibility.com (A.G.); g.sturaro@bci-biocompatibility.com (G.S.); 2 Department of Medicine and Health Sciences ‘V. Tiberio’, University of Molise, 86100 Campobasso, Italy; alessio.lepore@unimol.it (A.L.); alessia.arcaro@unimol.it (A.A.); gentilefabrizio@unimol.it (F.G.); alfonso.dicostanzo@unimol.it (A.D.C.); 3 Molise Regional Health Authority (ASREM), 86100 Campobasso, Italy

**Keywords:** αGal, gangliosides, neuroinflammation, Alzheimer’s biomarker, antibody pattern, diagnostic tool

## Abstract

Human anti-αGal antibodies (Abs), known for their marked polyreactivity, have been detected bound to the gray matter of the brains of Alzheimer’s disease (AD) patients, although their targets were unclear. Since αGal is a strictly xenogenic antigen absent in humans, this observation raised questions regarding the nature of the structures recognized by these antibodies. In this study, we investigated their potential interaction with gangliosides—glycan structures that are highly abundant in the central nervous system. Using a competitive inhibition ELISA, serum profiles of anti-αGal Abs isotypes and their indirect cross-reactivity with selected soluble gangliosides were analyzed in AD patients and healthy subjects (HSs). AD patients showed reduced levels of anti-αGal IgG and IgM, but increased IgA compared to HSs. Notably, pre-incubation with GM1, GM2, or GD1b did not reduce αGal–HSA binding in HS sera. In contrast, in AD sera, pre-incubation with GD1b reduced residual αGal–HSA binding for all antibody isotypes; additionally, GM1 inhibited IgM binding, and GM2 inhibited IgA binding. These results should therefore be interpreted as competitive inhibition patterns consistent with ganglioside-associated cross-reactivity rather than as direct evidence of antibody binding to immobilized gangliosides. Overall, the findings provide preliminary evidence that, in AD sera, a fraction of αGal–HSA-reactive antibodies can be competitively inhibited by selected gangliosides. This observation supports the presence of an altered humoral anti-carbohydrate signature in AD and identifies neuronal gangliosides as plausible candidate autologous targets that may help explain the previously reported binding of anti-αGal Abs to gray matter. However, given the indirect nature of the assay, these data should be considered hypothesis-generating and require confirmation by direct binding approaches.

## 1. Introduction

Alzheimer’s disease (AD) is a progressive neurodegenerative disorder characterized by memory loss, cognitive decline, and behavioral changes, primarily affecting the elderly. Hallmark pathological features include extracellular amyloid-beta (Aβ) plaques, intracellular neurofibrillary tangles composed of hyperphosphorylated tau protein, synaptic loss, and widespread neuronal degeneration. While AD has long been conceptualized primarily as a proteinopathy driven by Aβ and tau aggregation [1], this amyloid–tau-centered view is now increasingly integrated with a broader neuroimmune model in which innate and adaptive immune responses, vascular dysfunction, peripheral inflammation, and impaired blood–brain barrier (BBB) integrity actively contribute to disease onset and progression [2,3,4,5,6,7,8].

Within this inflammatory framework, chronic neuroinflammation is not merely considered a downstream consequence of neuronal damage but a dynamic process involving microglia, astrocytes, endothelial cells, peripheral immune cells, and soluble inflammatory mediators [2,3,4,5,6,7,8]. In particular, increasing attention has been directed toward the contribution of adaptive immunity to AD pathophysiology. B lymphocytes may participate in neuroinflammation through antigen presentation, cytokine secretion, antibody production, and interaction with T cells and microglia. Although their role in AD remains complex and context-dependent, recent evidence indicates that peripheral immune cells, including B lymphocytes, can influence neuroinflammatory cascades and may contribute either to protective immune surveillance or to chronic inflammatory damage [9]. In this broader context, studies in other neurodevelopmental and neuroinflammatory conditions have shown that B cells can display an imbalance between pro-inflammatory and anti-inflammatory cytokine profiles, with increased IL-6 and TNF-α and reduced IL-10 production, supporting the concept that B-cell functional polarization may be relevant across different central nervous system (CNS)-associated immune disorders [10]. Although such findings are not specific to AD, they reinforce the rationale for investigating disease-associated remodeling of humoral immune responses in neurological diseases.

Antibodies (Abs) have been detected in the CNS of AD patients, suggesting an active humoral immune component. The specificity and pathogenic relevance of these Abs remain incompletely understood, but their presence implies either increased CNS access of circulating immunoglobulins, local immune activation, or a breach of immune tolerance toward CNS-associated antigens. BBB dysfunction, endothelial activation, and choroid plexus alterations may facilitate the entry of circulating immune mediators and Abs into the CNS, thereby amplifying neuroinflammatory pathways [2]. Recent studies have further implicated the gut microbiota and peripheral immune priming as contributors to neuroinflammation, particularly in the context of intestinal barrier impairment, circulating bacterial products such as lipopolysaccharide, endothelial dysfunction, and BBB permeability [11]. This microbiota–gut–brain axis is especially relevant to natural and microbiota-induced Abs repertoires, including anti-carbohydrate Abs.

The αGal epitope (Galα1-3Galβ1-4GlcNAc-R) is a sugar moiety attached to the terminal part of glycoproteins and glycolipids present on the surfaces of various microorganisms and in all mammalian tissues except for Old World monkeys, apes, and humans. This epitope is generated by the α1,3-galactosyltransferase (α1,3GT) encoded by the GGTA1 gene. In humans, this gene underwent evolutionary mutations, leading to its silencing, resulting in the production of Abs directed against the αGal epitope. Approximately 1–3% of circulating Abs are represented by anti-αGal immunoglobulins, mainly IgG, IgM, and IgA classes. The presence of this pool of Abs against αGal is attributed to chronic exposure of the immune system to αGal-bearing microorganisms from the gut flora and has been considered a positive factor in human evolution, as it contributes to defense against pathogens expressing αGal [12,13].

Notably, anti-αGal Abs are intrinsically polyreactive, and under pathological conditions their specificity may broaden. Altered anti-αGal Abs repertoires have been described in autoimmune and inflammatory diseases, including Crohn’s disease, eosinophilic esophagitis, and IgA nephropathy, where these Abs display atypical isotype distribution, affinity, and glycan recognition profiles [14,15,16]. In AD, altered circulating levels of anti-Gal immunoglobulins have also been reported, with decreased IgG and IgM and increased IgA in patients compared with healthy subjects (HSs), suggesting a possible disease-associated shift in anti-Gal humoral immunity [17]. These changes may reflect altered host–microbiota interactions, chronic mucosal immune stimulation, class-switching phenomena, or selective consumption/redistribution of specific anti-carbohydrate Abs in inflammatory tissues.

In neuroinflammatory settings, anti-αGal Abs have been detected bound to gray matter in AD brains and in the cerebrospinal fluid of patients with multiple sclerosis, Guillain–Barré syndrome, and meningitis [7,18,19,20], suggesting that structural or contextual changes may enable recognition of endogenous CNS glycoconjugates. This possibility is particularly relevant in AD, where neurodegeneration, oxidative stress, Aβ deposition, tau pathology, and membrane remodeling may expose or alter glycan-containing structures that are normally poorly accessible to circulating Abs. Moreover, serum anti-glycosphingolipid Abs, including anti-ganglioside reactivities, have previously been reported in dementia and AD, supporting the hypothesis that humoral responses against neuronal membrane glycoconjugates may occur in neurodegenerative conditions [21].

Gangliosides (GLSs) are sialylated glycosphingolipids highly enriched in neuronal membranes and lipid rafts, where they regulate synaptic signaling, membrane organization, receptor function, and interact directly with Aβ peptides. Although GLSs do not contain canonical αGal epitopes, they present Gal- and GalNAc-containing glycan motifs that may become immunologically accessible under pathological conditions, particularly when clustered, oxidatively modified, or conformationally altered by Aβ binding [21]. In AD, such membrane and glycan remodeling may favor molecular mimicry or cross-reactivity between anti-αGal Abs and selected brain GLSs, thereby linking microbiota-driven humoral immunity with CNS-directed autoimmune or para-autoimmune mechanisms.

For the present exploratory study, GM1, GM2, and GD1b were selected as representative neuronal gangliosides because they differ in carbohydrate headgroup composition and degree of sialylation, are implicated in neuronal membrane organization and Aβ-associated processes, and provide a biologically relevant panel for evaluating potential anti-αGal antibody cross-reactivity.

This study aims to test the hypothesis that in AD, the anti-αGal Abs repertoire undergoes disease-specific remodeling, enabling cross-reactivity with selected brain GLSs. Using ELISA-based assays, we evaluated the distribution of anti-αGal Abs and their competitive inhibition profiles with GM1, GM2, and GD1b GLSs in sera from AD patients and HSs. By integrating anti-αGal serology with ganglioside-binding profiles, this work seeks to clarify whether anti-carbohydrate Abs may contribute to the humoral immune signature of AD and represent potential biomarkers or mechanistic mediators of neuroinflammatory damage.

## 2. Results

Before presenting the quantitative results, it is important to clarify the ELISA strategy used in this study. The assay was designed as a competitive inhibition ELISA to evaluate whether antibodies reactive toward the αGal–HSA conjugate also display cross-reactivity with selected soluble GLSs. In this format, the microtiter plate was coated with αGal–HSA, consisting of the αGal trisaccharide epitope Galα1-3Galβ1-4GlcNAc-R conjugated to human serum albumin as carrier protein. Serum samples were analyzed either directly, to quantify total αGal–HSA-reactive IgG, IgM, or IgA, or after overnight pre-incubation with soluble GM1, GM2, or GD1b.

**Figure 1:**
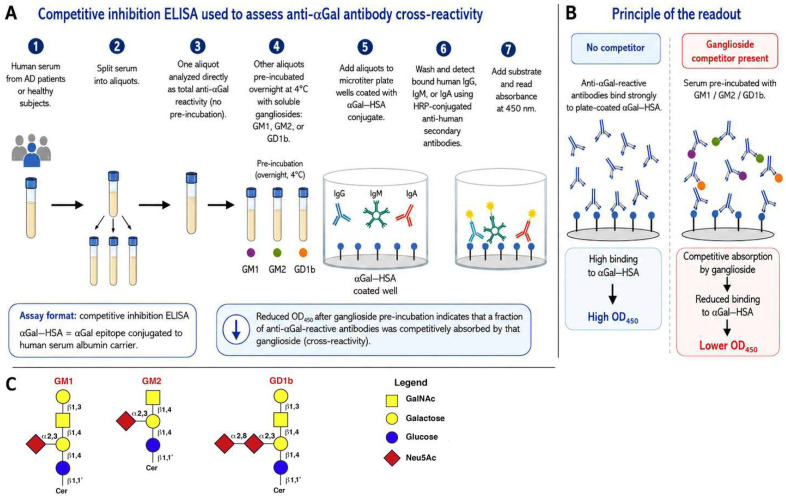
Competitive inhibition ELISA used to assess anti-αGal/HSA antibody cross-reactivity with selected GLSs. (**A**) Assay workflow. Human serum samples from AD patients and healthy subjects were divided into aliquots. One aliquot was analyzed directly to determine total αGal–HSA-reactive IgG, IgM, or IgA. Parallel aliquots were pre-incubated overnight at 4 °C with soluble GM1, GM2, or GD1b and then added to microtiter wells coated with αGal–HSA. Bound human IgG, IgM, or IgA was detected using HRP-conjugated anti-human secondary antibodies, and absorbance was read at 450 nm. (**B**) Principle of the readout. In the absence of a soluble GLS competitor, αGal–HSA-reactive Abs bind to the plate-coated αGal–HSA conjugate, generating a higher OD450 signal. If soluble GLSs competitively absorb a fraction of these Abs during pre-incubation, fewer Abs remain available to bind αGal–HSA, resulting in a lower OD450 signal. (**C**) Simplified structural formulas of the GM1, GM2, and GD1b gangliosides considered in the analyses.

Therefore, the assay does not directly measure binding of Abs to immobilized GM1, GM2, or GD1b. Instead, it measures the reduction in Abs binding to plate-coated αGal–HSA after soluble GLS pre-incubation. A decrease in OD450 indicates that a fraction of αGal–HSA-reactive Abs was competitively absorbed by the soluble GLS, suggesting cross-reactivity between αGal–HSA-reactive Abs and GLS. For clarity, the term “ganglioside-binding fraction” is used in the Results section to indicate the proportion of αGal–HSA-reactive Abs whose binding to αGal–HSA was inhibited by pre-incubation with a given GLS. The assay principle, workflow, and simplified structural formulas of the GM1, GM2, and GD1b gangliosides considered in the analyses are summarized in Figure 1.

**Figure 2:**
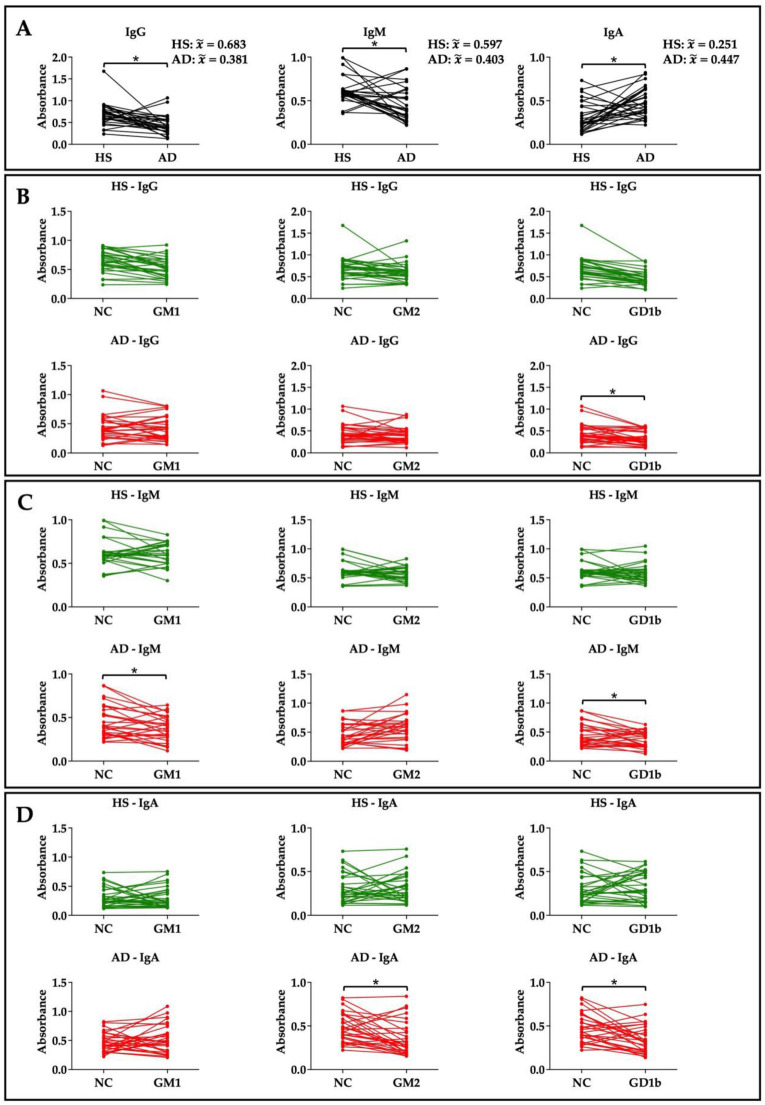
Paired analysis of αGal–HSA-reactive antibodies before and after ganglioside pre-incubation. Serum levels of αGal–HSA-reactive antibodies, expressed as absorbance at 450 nm (OD450), were evaluated in HSs and AD patients (*n* = 30 per group). (**A**) Total αGal–HSA-reactive IgG, IgM, and IgA levels in HSs and AD patients. (**B**–**D**) Paired comparison of total αGal–HSA-reactive antibody reactivity measured in the absence of gangliosides (NC = non-competitor) and the residual αGal–HSA binding after pre-incubation with soluble GM1, GM2, or GD1b. Panel B shows IgG, C shows IgM, and D shows IgA responses. Green lines represent HSs and red lines represent AD patients. Each line connects measurements obtained from the same individual before (NC) and after ganglioside pre-incubation, illustrating the change in αGal–HSA reactivity induced by each ganglioside. Statistical significance was assessed after evaluation of data normality using the Shapiro–Wilk test. Comparisons between HSs and AD patients were performed using an unpaired *t*-test or Mann–Whitney U test, as appropriate. Within-subject comparisons between NC and ganglioside-treated conditions were performed using a paired *t*-test or Wilcoxon signed-rank test, according to the distribution of the paired differences. * Statistical significance was defined as *p* < 0.05.

Considering the total expression of the different anti-αGal Abs isotypes, the previously reported findings are further confirmed [16]. There is a lower production of circulating Abs of the IgG and IgM type in subjects affected by AD compared to HSs; conversely, the IgA isotype is more highly expressed in AD patients (Figure 2A). However, given the exploratory nature of the study and the limited sample size, these findings should be interpreted as preliminary and descriptive of the present cohort rather than as definitive evidence of a generalized serological pattern in AD.

Regarding the ability of the various αGal–HSA-reactive antibody isotypes to be competitively inhibited by soluble GLSs (GM1, GM2, and GD1b), a significant difference was found between HSs and AD patients within the analyzed cohort. As described above, this assay does not quantify direct binding to immobilized GLSs; rather, it estimates the fraction of αGal–HSA-reactive Abs whose binding to αGal–HSA is reduced after pre-incubation with soluble GM1, GM2, or GD1b.

Notably, in HSs, none of the considered isotypes are able to interact with the GLSs (Figure 2B–D, HS green), indicating that, in the control group, soluble GLSs did not competitively absorb detectable fractions of αGal–HSA-reactive antibodies. This suggests that αGal–HSA-reactive Abs from HSs retained preferential reactivity toward the αGal–HSA ligand under the experimental conditions used.

In contrast, in AD patients, pre-incubation with GD1b significantly reduced residual IgG binding to plate-coated αGal–HSA (Figure 2A,B,D red). A schematic overview is provided in the Supplementary Material (Figure S1).

**Figure 3:**
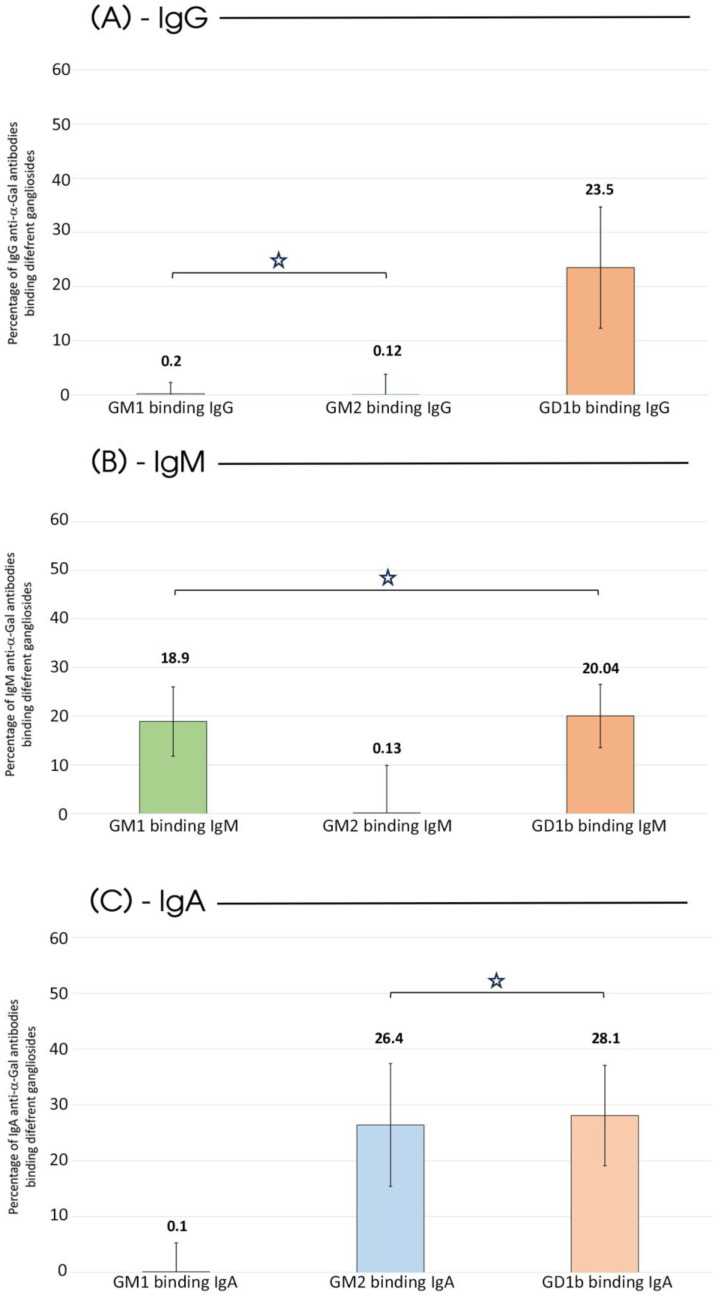
AD serum: GLS-mediated competitive inhibition of αGal–HSA-reactive antibodies. Percentage of GLS-inhibited αGal–HSA-reactive antibodies calculated by comparing residual OD450 after soluble GLS pre-incubation with the corresponding non-pre-incubated serum aliquot for each antibody isotype in AD patients. (**A**) IgG, (**B**) IgM, (**C**) IgA. Green bars represent binding to GM1 ganglioside, blue bars to GM2 ganglioside, and orange to GD1b gangliosides. As per Figure 1, *n* = 30. The white star identifies a non-statistically significant difference (*p* > 0.05, two-sided Student’s *t*-test). These values represent indirect estimates of competitive absorption/cross-reactivity and should not be interpreted as direct binding values obtained from ganglioside-coated plates. Vehicle controls were included to exclude nonspecific effects due to serum dilution or ethanol exposure.

This reduction indicates that approximately 23.5% of the αGal–HSA-reactive IgG fraction was competitively absorbed by soluble GD1b under the experimental conditions used, providing indirect evidence consistent with IgG cross-reactivity with GD1b (Figure 3A).

The competitive inhibition pattern was also evident for the IgM and IgA isotypes. Pre-incubation with GM1 and GD1b reduced residual IgM binding to αGal–HSA, corresponding to an estimated 18.9% and 20.04% ganglioside-inhibited fraction, respectively (Figure 3B). Similarly, pre-incubation with GM2 and GD1b reduced residual IgA binding to αGal–HSA, corresponding to an estimated 26.4% and 28.1% ganglioside-inhibited fraction, respectively (Figure 3C). These values should therefore be interpreted as the percentage of αGal–HSA-reactive Abs competitively absorbed by the corresponding soluble ganglioside rather than as direct binding values obtained from ganglioside-coated plates. Thus, the data support an indirect competitive inhibition pattern compatible with GLS cross-reactivity, but they do not by themselves demonstrate direct Abs binding to GM1, GM2, or GD1b.

Finally, in addition to the fact that all isotypes of AD patients have a similar affinity for the GD1b GLS, a clear pattern emerges in which anti-αGal IgM recognizes GM1 and IgA recognizes GM2 (Table 1). Thus, Table 1 summarizes the isotype-specific ganglioside-inhibition pattern observed in the competitive ELISA rather than direct antibody binding to immobilized GLSs. For this reason, the terms “binding” or “recognition” are used cautiously and only as indirect interpretations of competitive inhibition, not as direct binding measurements.

## 3. Discussion

The aim of this study was to investigate whether the anti-αGal Abs repertoire in AD shows disease-associated remodeling and whether these antibodies can recognize selected brain GLSs as potential autologous glycan targets. The main findings were as follows: (i) AD patients displayed an altered circulating anti-αGal Abs isotype profile, characterized by lower IgG and IgM levels and higher IgA levels compared with HSs; (ii) pre-incubation with the tested GLSs did not detectably inhibit αGal–HSA binding in HS sera; and (iii) AD sera showed an isotype-specific GLS-mediated competitive inhibition pattern involving GD1b, GM1, and GM2. In particular, GD1b was recognized by all antibody isotypes, whereas GM1 and GM2 showed preferential binding by IgM and IgA, respectively. These observations support the presence of an altered humoral anti-carbohydrate signature in the analyzed AD cohort and provide a rationale for exploring GLSs as plausible targets of anti-αGal Abs cross-reactivity.

It is important to emphasize that these findings derive from a competitive inhibition ELISA and therefore provide indirect evidence of ganglioside-associated cross-reactivity. The observed reduction in αGal–HSA binding after soluble GLS pre-incubation indicates that a fraction of αGal–HSA-reactive Abs was no longer available to bind the plate-coated antigen. Nevertheless, the inhibition profile was not randomly distributed across all isotypes and GLSs; rather, it showed a selective pattern in AD sera and was not detected in HS sera under the same experimental conditions. This selectivity argues against a purely nonspecific measurement fluctuation, although direct binding and kinetic studies will be necessary to confirm the molecular interaction.

Before interpreting these findings in a biological framework, it is important to emphasize their exploratory nature. The present study included a relatively small cohort of AD patients and healthy subjects, which limits statistical power and increases the risk that some observed differences may be cohort-specific. Moreover, the sample size does not allow robust subgroup analyses according to disease severity, sex, age, comorbidities, medication use, blood group, or other potentially relevant clinical variables. In addition, the lack of neurological disease-control groups, such as patients with other neurodegenerative diseases or non-AD cognitive decline, prevents us from determining whether the observed anti-αGal/ganglioside reactivity is specific to AD or reflects a broader feature of aging, cognitive impairment, or neuroinflammation. Therefore, the term “AD-associated” is used cautiously, and future studies will include additional disease-control cohorts to address this issue. Therefore, the statistical results should be interpreted as hypothesis-generating rather than confirmatory.

As the molecular landscape of AD becomes increasingly defined, membrane lipids emerge as active contributors rather than passive components. Among these, glycosphingolipids are highly abundant in the CNS, where they represent a significant portion of neuronal plasma membrane lipids [21]. Their amphipathic structure, enriched with terminal sialic acid residues, allows them to assemble into lipid rafts, specialized membrane microdomains that play key roles in signal transduction, synaptic plasticity, and membrane stability [21,22]. This concept is supported by recent reviews showing that GLSs are essential regulators of neuronal differentiation, synaptic organization, receptor activity, membrane trafficking, and neuroinflammatory responses, and that alterations in GLS metabolism are increasingly associated with major neurological and neurodegenerative diseases [23,24,25].

**Figure 4:**
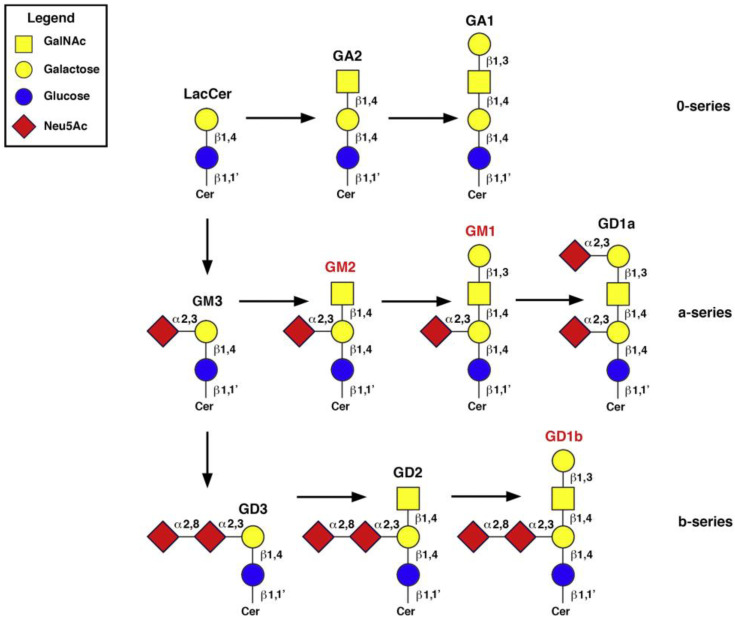
Structural comparison of the different GLSs. Chemical/morphological evolution of the different GLSs belonging to the 0, a, and b series.

The GLSs selected in this study (GM1, GM2, and GD1b) were chosen because they provide a focused representation of neuronal membrane GLSs with different carbohydrate headgroups and sialylation patterns. This is relevant because the carbohydrate moiety exposed at the membrane surface determines both protein–lipid interactions and potential antibody recognition. Structurally, although GM1, GM2, and GD1b lack canonical αGal motifs, they display repeating Gal/GalNAc residues that can mimic αGal-like epitopes when clustered in lipid rafts (Figure 4).

The rationale for including each GLS was therefore specific. GM1 was selected because it is one of the best-characterized neuronal GLSs, is enriched in lipid raft-like microdomains, and has been repeatedly implicated in Aβ binding, aggregation, and formation of GM1-bound Aβ complexes, which may act as endogenous seeds for amyloid assembly [26,27]. GM2 was included because altered GM1 and GM2 distribution has been reported in AD cortical tissue and because GM2 contains GalNAc/Gal motifs that may be relevant for evaluating glycan-dependent antibody cross-reactivity [28]. GD1b was selected as a representative disialoganglioside of neuronal membranes; its more complex sialylated headgroup allows comparison with monosialylated GLSs and helps assess whether anti-αGal Abs reactivity is influenced by ganglioside structure and sialylation. Together, these three molecules provide a biologically meaningful but limited panel to explore whether anti-αGal Abs may recognize selected neuronal GLSs under AD-associated conditions.

During neurodevelopment, GLSs orchestrate axonal growth, synaptogenesis, and neuronal differentiation. Some species also exhibit intrinsic neuroprotective properties and bind Aβ in a conformation-dependent manner, thereby modulating its aggregation behavior [23,29,30]. Among these molecules, GM1 plays a pivotal role in the earliest stages of Aβ aggregation. Monomeric Aβ preferentially associates with negatively charged GLSs, particularly GM1 [31,32], as well as with membranes enriched in GM1, sphingomyelin, and cholesterol, which closely resemble neuronal lipid rafts [33]. Several biochemical, biophysical, and membrane-model studies have shown that Aβ–ganglioside interactions can promote Aβ conformational transitions, membrane-associated aggregation, and the formation of GM1-bound Aβ species, which have been proposed as endogenous seeds for amyloid assembly [26,34]. Biochemical data indicate that the affinity of Aβ (1–40) for ganglioside-containing membranes follows the order GT1b < GM1 < GD1b < GD1a [35], underscoring the importance of GM1-rich domains as preferential docking platforms.

Further support for the immunological relevance of GLSs comes from studies reporting anti-ganglioside Abs in dementia and AD. Anti-GM1 antibodies were described in patients with Alzheimer’s disease and other dementias, and later studies suggested that elevated anti-GM1 IgM may correlate with older age and more severe cognitive impairment in demented patients [36,37,38]. In addition, anti-glycosphingolipid Abs, including anti-GQ1bα/anti-Chol-1 reactivity, have been reported in AD and vascular dementia, although not all studies support their utility as robust biomarkers [21,39]. These data do not establish causality, but they strengthen the plausibility that humoral responses against neuronal glycolipids may occur in a subset of neurodegenerative conditions.

**Figure 5:**
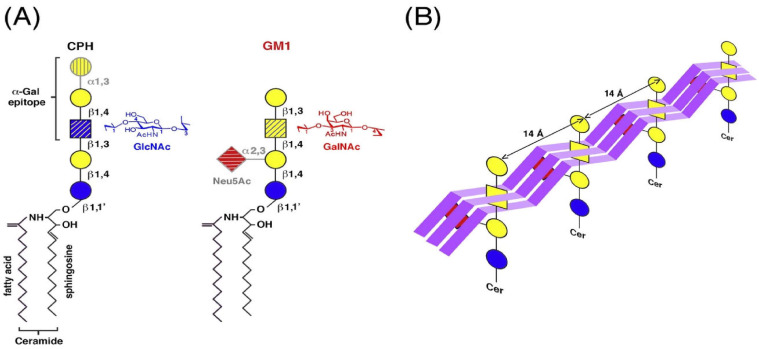
Structural comparison between CPH and GM1 and their interaction with Aβ aggregates. (**A**) Structural differences between ceramide pentahexoside (CPH), a neutral glycosphingolipid bearing the α1-3Gal epitope, and GM1. Differences are highlighted: (a) the galactose residue at the reducing end of CPH (yellow circle with gray cross-hatch), which is missing in GM1; (b) the N-acetylglucosamine residue of CHP (blue square with yellow cross-hatch and blue formula), where carbon 3 bears a –OH group and carbon 4 is engaged in a β1-4 bond with galactose on the reducing end side, as opposed to the N-acetylgalactosamine residue of GM1 (yellow square with blue cross-hatch and red formula), where carbon 4 bears a –OH group and carbon 3 is engaged in a β1-3 bond with galactose on the reducing end side; (c) the sialic acid (Neu5Ac) residue in GM1 (red diamond with gray cross-hatch), which is missing in CPH. (**B**) A string array of GM1 molecules in a cell membrane interacting with a β-sheet-rich Aβ oligomer/fibril through bonds at regular intervals of 14 Å between amino acid residues of Aβ peptides and Neu5Ac and GalNAc residues of GM1 molecules. It is suggested that Neu5Ac and GalNAc residues could be masked in whole or in part, respectively, by interacting with the Aβ fibrils. Refer to Figure 4 for the symbolization of carbohydrate residues.

Aβ–ganglioside interactions provide a biologically plausible context for the observed cross-reactivity, but they should not be interpreted as direct evidence of the mechanism underlying our serological findings. Previous biochemical and membrane-model studies have shown that GM1-enriched lipid raft-like domains can favor Aβ binding, conformational transition, and aggregation [26,32,33,35,40,41,42,43,44,45]. These interactions may alter the spatial exposure of GLS carbohydrate headgroups, potentially generating glycan arrangements that differ from those present under physiological conditions. In this framework, anti-αGal/ganglioside cross-reactivity could reflect AD-associated membrane remodeling or Aβ–ganglioside complex formation. This concept is schematically illustrated in Figure 5, which compares the αGal-bearing ceramide pentahexoside structure with GM1 and proposes how Aβ interaction with GM1-rich membrane domains may modify the accessibility of specific carbohydrate residues. Although GM1 differs from ceramide pentahexoside (CPH), which carries a canonical α1–3Gal epitope (Figure 5A), its headgroups can undergo functional reorganization when bound to Aβ oligomers or fibrils (Figure 5B). Direct peptide interaction may mask sialic acid and, partially, the GalNAc residues, leaving galactose moieties exposed in spatial arrangements that mimic αGal-like epitopes despite lacking a true Galα1–3Gal linkage. This Aβ-induced glycan reshaping potentially provides a reasonable structural basis for the generation of antibodies against Aβ–ganglioside complexes that can cross-react with α1–3Gal-bearing glycolipids. Such cross-reactivity may therefore serve as a molecular footprint of membrane-level Aβ aggregation.

However, the present study did not directly assess Aβ–ganglioside complexes, membrane remodeling, or antibody binding in brain tissue; therefore, this interpretation remains hypothetical.

In parallel, anti-GLS Abs have been implicated in neurodegenerative and immune-mediated neurological disorders. Elevated anti-GM1 Abs have been reported in demented patients [36,37], anti-GD1 Abs can affect axonal regeneration in experimental models [46], and anti-GLS Abs are frequently detected in autoimmune neuropathies such as GBS and chronic inflammatory demyelinating polyneuropathy [47,48]. These studies support the general concept that GLSs can become targets of humoral immune responses in neurological diseases, although they do not establish that such antibodies are pathogenic in AD. Within this broader context, our study suggests that, in the analyzed AD cohort, anti-αGal Abs display an isotype-specific cross-reactivity toward selected GLSs, particularly GD1b, GM1, and GM2. This pattern was not detected in the HS group analyzed in this study. However, this observation should not be overgeneralized, and larger independent cohorts are required before defining this profile as a reproducible AD-associated signature.

Physiologically, anti-αGal Abs arise in response to gut microbiota and environmental exposure, with specificity primarily directed toward Galα1–3Gal. Chronic inflammation and autoimmune conditions may broaden antibody recognition through mechanisms such as affinity maturation or epitope spreading [14,49,50,51]. Similar alterations of anti-αGal Abs reactivity have been described in Crohn’s disease, eosinophilic esophagitis, and IgA nephropathy [15,16]. In AD, the altered anti-αGal Abs isotype distribution and ganglioside-binding profile observed here may therefore reflect remodeling of the humoral anti-carbohydrate repertoire. Nevertheless, the present data do not allow us to determine whether this remodeling results from chronic immune stimulation, antibody consumption, mucosal immune activation, or other mechanisms.

Taken together, the present findings indicate that AD patients in this exploratory cohort display an altered anti-αGal Abs humoral profile and an isotype-specific GLS-mediated competitive inhibition pattern. These are the main data-supported conclusions of the study. The proposed links with Aβ–ganglioside remodeling, BBB dysfunction, complement activation, and local neuroinflammation remain biologically plausible but speculative.

Human anti-αGal Abs possess heterogeneous paratopes tolerant to structural variation around the Gal/GalNAc core, explaining documented cross-reactivity with de-fucosylated B-blood group determinants [52,53,54,55]. In AD brains, altered GLSs distribution may intensify this effect. GM1 and GM2 accumulate in affected cortical regions [31,32], promoting Aβ aggregation into GAβ complexes that cluster glycolipid headgroups and enhance antibody avidity [23,31,56]. Conversely, GD1b levels decline in correlation with disease severity [31,33]. These lipid alterations may facilitate amyloid pore formation and neurotoxicity [40].

Recent reviews support the concept that BBB dysfunction in AD is closely linked to vascular injury, neuroinflammation, impaired clearance mechanisms, and altered communication between peripheral immunity and the CNS [57,58,59]. Thus, BBB dysfunction could represent a permissive condition through which circulating antibodies or immune complexes may gain access to CNS-associated targets. If anti-αGal/ganglioside-reactive antibodies were present within CNS tissue, they could theoretically participate in local immune amplification through complement- or Fc-receptor-related pathways, both of which have been implicated in AD neuroinflammation [60,61]. Nevertheless, our data remain limited to serum antibody reactivity and do not demonstrate CNS penetration, complement deposition, Fc receptor activation, or antibody-mediated neuronal injury. Therefore, these mechanisms should be considered speculative and are presented only as possible directions for future investigation.

Lectin histochemistry reinforces the biological plausibility of this hypothesis: amyloid plaques stain intensely with lectins recognizing galactose, GalNAc, GlcNAc, and sialic acid, and Griffonia simplicifolia IB4, specific for terminal αGal, binds glycoconjugates within plaques [12,62,63], supporting the presence of accessible αGal-like motifs [64].

Physiological parallels highlight the mechanistic relevance of this mechanism. In aging erythrocytes, exposure of neolacto-series glycolipid X_2_ enables anti-αGal-mediated clearance [65,66,67,68,69], illustrating the natural role of these antibodies in glyco-immune homeostasis. By analogy, one possible interpretation is that AD-related membrane remodeling and glycan exposure may secondarily promote anti-αGal/ganglioside cross-reactivity. Alternatively, these Abs could contribute to local inflammatory amplification once tissue damage and barrier dysfunction have already occurred. At present, the directionality of this relationship cannot be determined, and the observed antibody pattern may reflect either a contributor to pathology, a downstream consequence of neurodegeneration, or an epiphenomenon of systemic immune dysregulation.

The possibility that anti-αGal Abs may recognize structurally related or conformationally clustered glycan motifs is also consistent with recent structural studies of the human anti-α-galactosyl antibody response. Langley et al. showed that specific genetic and structural determinants shape the human anti-αGal Abs response yet also exhibit molecular features consistent with heterogeneous antigen recognition [54]. This supports the rationale that, under pathological conditions characterized by membrane remodeling, glycan clustering, or altered exposure of Gal/GalNAc-containing structures, anti-αGal Abs may show broader glycan reactivity. In the present study, this concept remains hypothetical, but it provides a mechanistic framework for interpreting the observed binding to GM1, GM2, and GD1b.

Within this model, anti-αGal Abs should primarily be considered, at this stage, as a disease-associated humoral signature and as candidate blood-based biomarkers of AD. Their isotype-specific reactivity toward GM1, GM2, and GD1b offers translational potential for minimally invasive diagnostics and provides a rationale for future mechanistic studies aimed at determining whether these antibodies have pathogenic, protective, or merely secondary roles [17,47,48,49].

In this framework, AD emerges not solely as a disorder of protein aggregation, but as a disease in which membrane lipid dynamics and glyco-immunological responses intersect—opening new perspectives on both pathogenesis and clinical monitoring. Nevertheless, causal relationships between anti-αGal Abs remodeling and AD progression cannot be inferred from the present data, and will require larger longitudinal cohorts, CSF analyses, neuropathological validation, and functional assays.

### Limitations

This study has several limitations that should be considered when interpreting the findings. First, the relatively small sample size (30 AD patients and 30 HSs) and the exploratory nature of this study limit its statistical power and the generalizability of the results. These characteristics also preclude detailed subgroup analyses according to the disease stage, treatment status, or other clinically relevant variables.

Although sample collection was standardized with respect to timing (morning collection), fasting status, and handling procedures, and exclusion criteria were applied to reduce potential confounding factors, residual confounding cannot be completely excluded. Variables such as age, sex, medication use (e.g., anti-inflammatory or immunomodulatory drugs), and underlying comorbidities (e.g., metabolic or autoimmune disorders) may influence Abs levels. While relatively homogeneous selection criteria for AD patients and HSs were adopted to minimize such variabilities, these factors remain potential sources of bias and should be more comprehensively addressed in larger, stratified cohorts.

A further limitation is the cross-sectional design, which prevents the evaluation of longitudinal changes in Abs profiles and their relationship with cognitive decline over time. Importantly, this design does not allow causal inferences. Consequently, it remains unclear whether the observed anti-αGal/ganglioside reactivity actively contributes to AD-related neuroinflammatory or neurodegenerative processes, represents a secondary consequence of membrane remodeling, neuronal damage, or BBB dysfunction, or simply reflects broader systemic immune alterations.

Another limitation is the absence of CSF analyses, which would have provided more direct information regarding intrathecal Abs activity and immune responses within the central nervous system.

Therefore, the present findings should be regarded as preliminary and hypothesis-generating. Future studies involving larger, longitudinally followed cohorts will be necessary to explore potential associations between anti-αGal Abs patterns and cognitive measures.

Overall, the present data provide an initial exploratory observation of altered GLS-mediated competitive inhibition of αGal–HSA-reactive antibodies in AD. However, larger multicenter studies with adequate statistical power, correction for potential confounders, independent validation cohorts, and direct binding confirmation using ganglioside-based ELISA or biophysical approaches will be necessary before these findings can be considered biologically and clinically generalizable.

## 4. Materials and Methods

### 4.1. Patients and Healthy Subjects

The participants in the study (*n* = 60) were consecutively recruited at the Center for Research and Training in Medicine of Aging (CeRMA) of the University of Molise (Italy). The AD patients (*n* = 30) fulfilled the National Institute of Aging and Alzheimer’s Association (NIA-AA) diagnostic criteria for “probable AD with documented decline” [70]. Cognitive status was assessed using the Mini Mental State Examination (MMSE), a 30-point questionnaire evaluating orientation, memory, attention, language, and visuospatial skills [71]. Scores below 24 suggest cognitive impairment. Dementia severity was determined using the Clinical Dementia Rating (CDR), which assesses six domains of cognitive and functional performance [72]. A global score of 0 indicates no dementia, while scores of 0.5, 1, 2, and 3 correspond to very mild, mild, moderate, and severe dementia, respectively. The enrolled AD group scored < 24 on the MMSE and >0.5 on the CDR. Thus, the AD group included patients with clinically established cognitive impairment, and dementia severity was documented using standardized cognitive and functional measures. However, given the limited number of participants, patients were analyzed as a single AD group rather than being stratified according to CDR stage or MMSE severity.

To rule out other potential causes of cognitive impairment, all patients underwent blood tests (including full blood count, erythrocyte sedimentation rate, urea and electrolytes, thyroid function, vitamin B12, and folate) and brain imaging. Thirty sex/age-matched cognitively HSs were recruited as a control group. Since anti-αGal Abs levels can be altered in the context of different pathologies and treatments, subjects with pathologies, such as rheumatoid arthritis, interstitial cystitis, eosinophilic esophagitis [16], Henoch-Schönlein purpura, IgA nephropathy, Crohn’s disease and ulcerative colitis [18], or treated with anticancer medications such as Cetuximab (or Erbitux) [49], animal derived tissue patches, cartilaginous grafts, or bioprostheses such as biological heart valves [73], were excluded. The clinical and demographic characteristics of the two groups of participants are summarized in Table 2. In addition to age and sex, Table 2 reports education level, BMI, cognitive status (assessed by the MMSE), blood group distribution, major comorbidities, and ongoing medication use. These variables were included because demographic characteristics, disease stage, metabolic or vascular comorbidities, blood group, and pharmacological treatments may influence systemic immune status and circulating antibody profiles. Therefore, these factors were considered during data interpretation, although the exploratory sample size did not allow for robust stratified or multivariable analyses.

This study was conducted in accordance with the ethical principles stated in the Declaration of Helsinki and approved national and international guidelines for human research. The Institutional Review Board (IRB) of the University of Molise approved the study (IRB Prot. n. 007-08-2018). Written informed consent was obtained from either the participants or caregivers.

### 4.2. Blood Collection and Processing

Venous blood samples were collected between 8:00 and 8:30 AM, after an overnight fast of at least 8–10 h. Blood was drawn by standard venipuncture using a sterile single-use blood collection set and serum separator vacuum tubes (Vacutainer^®^, Becton & Dickinson, Milan, Italy). Serum was separated within 2 h by centrifugation at 2500× *g* for 10 min at 20 °C using an Eppendorf^®^ 5810 R centrifuge (Eppendorf SE, Hamburg, Germany). The serum was then carefully aliquoted into polypropylene tubes and stored at −80 °C until further use.

### 4.3. Competitive Inhibition ELISA Assay

Determination of the ability of different human αGal–HSA-reactive antibody isotypes (IgG, IgM, and IgA) to cross-react with soluble GLSs (GM1, GM2, GD1b) was performed in triplicate using a modified competitive inhibition ELISA test (patent EP2626701). This assay was not based on the direct immobilization of GM1, GM2, or GD1b on the plate. Instead, it evaluated whether soluble GLSs could competitively reduce the binding of serum αGal–HSA-reactive antibodies to plate-coated αGal–HSA.

The plate antigen consisted of αGal conjugated to human serum albumin (αGal–HSA; Dextra Laboratories, Berkshire, UK). The carbohydrate moiety corresponds to the αGal trisaccharide Galα1-3Galβ1-4GlcNAc-R, covalently linked to HSA as carrier protein. In the present assay, antibodies detected against this antigen are therefore operationally defined as αGal–HSA-reactive Abs. The term anti-αGal Abs is retained throughout the manuscript to maintain consistency with previous literature and the established use of αGal–HSA as a surrogate antigen for anti-αGal Abs detection.

GM1, GM2, and GD1b were selected as a targeted exploratory panel of neuronal gangliosides based on their abundance or relevance in neuronal membranes, their involvement in lipid raft organization and Aβ–membrane interactions, and their structurally distinct carbohydrate headgroups. This selection was intended to evaluate representative mono- and disialylated GLSs with potential immunological relevance rather than to provide a comprehensive screening of all brain GLS species.

Each patient’s serum was diluted 50-fold with Phosphate-Buffered Saline (PBS, Merck Life Science, Darmstadt, Germany) in a final volume of 2 mL GLSs, (GM1 and GD1b, Merck Life Science Cat. Nos. 345747 and 345742, respectively; GM2 BOC Sciences, Shirley, NY, USA CAS No. 19600-01-2), and was solubilized in absolute ethanol at a concentration of 125 µg/mL. Part of the sera was analyzed directly to evaluate differences in the total anti-Gal Abs content between HSs and AD patients. Conversely, to evaluate the specificity towards GLSs, serum aliquots were pre-incubated with soluble GLSs before exposure to the αGal–HSA-coated plate. Specifically, 20 µL of each GLS was added to 500 µL of patients’ serum (125 ug/mL) and incubated overnight at +4 °C. To control for possible dilution and solvent effects, matched vehicle-control aliquots were prepared for each serum sample by adding the same volume, 20 µL, of ethanol-containing solvent without GLS to 500 µL of serum and incubating the samples under identical conditions. Therefore, GLS-pre-incubated samples and vehicle-control samples had the same final dilution and ethanol exposure. The residual binding measured after GLS pre-incubation was compared with the corresponding vehicle/dilution-matched control rather than with an untreated serum aliquot alone. This control was included to exclude nonspecific reductions in OD450 caused by serum dilution or ethanol-mediated interference with antibody binding.

For each isotype, a Polysorp 96-well plate (Nunc, Rochester, NY, USA) was coated with 50 µL of αGal/human serum albumin (HSA) (Dextra Laboratories, Berkshire, UK, Product Code: NGP3334), 5 µg/mL, for 2 h at 37 °C.

After washing three times with PBS, the blocking procedure was performed using 300 µL per well of 2% HSA for 2 h at RT in the dark. The wells were then washed three times, as mentioned above. A set of wells was loaded with 100 µL of non-pre-incubated serum (total Abs), while parallel wells were loaded with serum aliquots pre-incubated with GM1, GM2, or GD1b. Plates were then incubated for 3 h at 37 °C. After washing, the appropriate horseradish peroxidase (HRP)-conjugate secondary antibody [1:100] was added: anti-human IgG, anti-human IgM, or anti-human IgA (Merck Lifescience, Darmstadt, Germany Cat. Nos. A0170, I0759, and I0884, respectively). Plates were then incubated for 1 h at 37 °C. After an additional washing step, 100 µL of HRP substrate buffer was added to each well and incubated for 5 min at RT in the dark. Absorbance was measured at 450 nm using a microplate spectrophotometer (Multiskan Sky, Thermo Fisher Scientific, Waltham, MA, USA).

For each isotype, the percentage of GLS-inhibited αGal–HSA-reactive Abs was calculated by comparing the OD450 value obtained after GLS pre-incubation with the OD450 value obtained from the corresponding non-pre-incubated serum aliquot. This value represents the fraction of αGal–HSA-reactive Abs whose binding to αGal–HSA was reduced by soluble GLS pre-incubation and was used as an indirect estimate of ganglioside cross-reactivity.

Intra-plate variability was assessed by calculating the coefficient of variation (CV) among technical replicates of the same sample. The mean intra-plate CV was 8.2%; values above this threshold were excluded from the analysis or repeated. Inter-plate variability was monitored using the same internal reference serum sample included in each plate. The mean inter-plate CV was below 13.7%. These values indicate that the observed GLS-mediated inhibition exceeded the expected technical variability of the assay.

### 4.4. Statistical Analysis

Statistical analyses were performed using a two-tailed *t*-test or one-way ANOVA test with Dunnett correction when comparing multiple groups to specific conditions (GraphPad Prism v.8 and 9). Statistical analyses were performed using a 95% confidence level. This standard threshold was applied throughout the study to assess the significance of differences observed between groups. Accordingly, *p*-values less than 0.05 were considered statistically significant. The statistical tests, replicate experiments, and *p*-values are all cited in the figures and/or figure captions. Statistical tests were justified as appropriate for every figure, and the data met the assumptions of the tests. The ranges of the x- and y-axes of scatter plots were determined to include all of the data points. The sample size for each experiment and the replicate number of experiments are included in the figure legends, as well as the specific test used for the analysis.

Data distribution was assessed using the Shapiro–Wilk normality test. For comparisons between HS and AD subjects, normality was evaluated separately within each group and antibody isotype. Normally distributed data were analyzed using an unpaired two-tailed Student’s *t*-test, whereas non-normally distributed data were analyzed using the Mann–Whitney U test. For within-subject comparisons before and after ganglioside pre-incubation, normality was assessed on the paired differences. Normally distributed paired differences were analyzed using a paired two-tailed *t*-test, whereas non-normally distributed paired differences were analyzed using the Wilcoxon signed-rank test. A *p*-value < 0.05 was considered statistically significant.

## 5. Conclusions

This study indicates that AD patients display an altered circulating anti-αGal Abs profile together with an isotype-specific GLS-mediated competitive inhibition pattern that was not observed in HSs. In particular, GD1b inhibited αGal–HSA-reactive IgG, IgM, and IgA in AD sera, whereas GM1 and GM2 showed more selective inhibition of IgM and IgA, respectively. These findings suggest that a fraction of αGal–HSA-reactive antibodies in AD sera may be associated with ganglioside-related cross-reactivity.

These observations should be interpreted with caution, as the competitive inhibition ELISA used in this study provides indirect evidence and does not demonstrate direct Abs binding to immobilized gangliosides. Therefore, the results should be considered preliminary and hypothesis-generating.

Overall, the present data support the possibility that remodeling of the anti-carbohydrate humoral response occurs in AD and may involve neuronal ganglioside-related targets. Future studies in larger, independent, and longitudinal cohorts, including CSF analyses and direct ganglioside-binding assays, will be required to validate these findings and to clarify whether anti-αGal/ganglioside-reactive Abs represent biomarkers, secondary immune signatures, or potential contributors to AD-related neuroinflammatory mechanisms.

## Figures and Tables

**Table 1:** Isotype-specific GLS-mediated competitive inhibition pattern of αGal–HSA-reactive antibodies in HS and AD sera. Note: “+” indicates detectable competitive inhibition of αGal–HSA binding after pre-incubation with the corresponding soluble GLS. “++” points to very strong competitive inhibition. The table does not indicate direct antibody binding to immobilized GM1, GM2, or GD1b.

		GM1	GM2	GD1b
HS	IgG	-	-	-
	IgM	-	-	-
	IgA	-	-	-
AD	IgG	-	-	+
	IgM	+	-	+
	IgA	-	++	++

**Table 2:** Demographic and clinical characteristics of the study groups.

Characteristics	AD (N. 30)	HSs (N. 30)	F (1,59)/X^2 †^	*p*
Females/males (N.)	19/11	15/15	1.086	0.297
Age (mean ± SD, y) (range, y)	83.77 ± 5.89 (70–96)	80.83 ± 6.04 (70–93)	3.631	0.062
Education level (mean + SD, y)	9.10 ± 5.27	11.77 ± 4.19	4.708	0.034
BMI (mean + SD, kg/m^2^)	24.37 ± 4.56	26.37 ± 3.70	3.475	0.067
MMSE (score)	17.88 ± 6.85	30.07 ± 1.31	91.632	<0.001
Blood group (N; %)				
0	13; 43.3%	14; 46.6%	0.067	0.795
A	13; 43.3%	8; 26.6%	1.832	0.176
B	3; 10%	6; 20%	1.176	0.278
AB	1; 3.3%	2; 6.6%	0.351	0.554
Medical History (N; %)				
Smoke *	3; 10%	3; 10%	0.000	1.000
Dyslipidemia	11; 36.6%	12; 40%	0.071	0.791
Diabetes	8; 26.6%	6; 20%	0.373	0.542
Hypertension	17; 56.6%	19; 63.3%	0.278	0.598
Myocardial infarction	3; 10%	3; 10%	0.000	1.000
TIA/Stroke	3; 10%	1; 3.33%	1.071	0.301
Drugs (N; %)				
Antihypertensive	17; 56.6%	18; 60%	0.069	0.793
Lipid-lowering	10; 30%	11; 36.6%	0.073	0.787
Hypoglycemic	8; 26.6%	6; 20%	0.373	0.542
Antiacid	12; 40%	11; 36.6%	0.071	0.791
Antiplatelet	13; 43.3%	12; 40%	0.069	0.793
Anti-inflammatory	3; 10%	3; 10%	0.000	1.000

* Current smoker; AD, Alzheimer’s disease; HSs, healthy subjects; BMI, body mass index; MMSE, Mini Mental State Examination; TIA, transient ischemic attack.; † as described in the Methods section, F pertains to the evaluation of age, schooling, BMI, and MMSE; X2 applies to all other parameters.

## Data Availability

The original contributions presented in this study are included in the article. Further inquiries can be directed to the corresponding author.

## Supplementary Materials

The following supporting information can be downloaded at: https://www.mdpi.com/article/10.3390/ijms27146190/s1.

## Abbreviations

The following abbreviations are used in this manuscript:

Abs	Antibodies
AD	Alzheimer’s Disease
CNS	Central Nervous System
BBB	Blood–Brain Barrier
CSF	Cerebrospinal fluid
GBS	Guillan Barré Syndrome
GLSs	Gangliosides
HS	Healthy Subjects
CerMA	Center for Research and Training in Medicine of Aging
NIA-AA	National Institute of Aging and Alzheimer’s Association
MMSE	Mini-Mental State Examination
CDR	Clinical Dementia Rating
PBS	Phosphate-Buffered Saline
ELISA	Enzyme-Linked Immunosorbent Assay
HSA	Human Serum Albumine
BMI	Body Mass Index
TIA	Transient Ischemic Attack
