# Supplementary material for: Anti-Alpha-Gal Antibodies Against Gangliosides: Preliminary Data on a New Autoimmune Target in Alzheimer’s Disease Patients
Source: Int J Mol Sci. 2026 Jul 10;27(14):6190. doi: 10.3390/ijms27146190 (PMC13410784; doi:10.3390/ijms27146190)
Supplement: Supplementary file 1 [file ijms-27-06190-s001.zip › ijms-4287039-supplementary.pdf]

Supplementary Materials

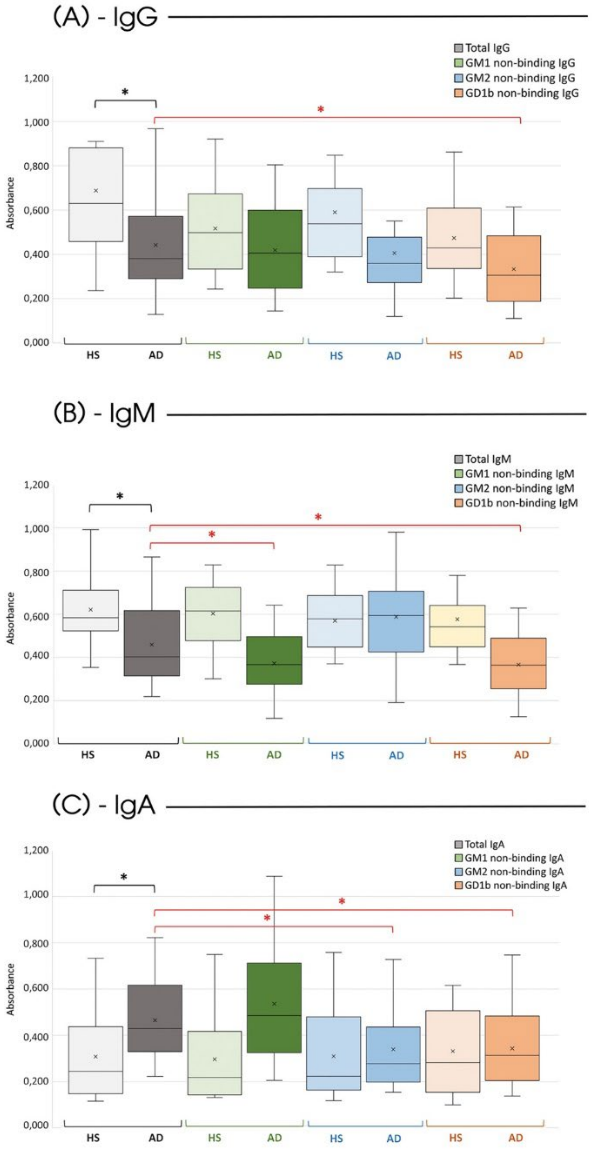

Figure S1. Serological quantification of αGal–HSA-reactive antibodies. Serum levels, expressed as absorbance at 450 nm (OD450), of αGal–HSA-reactive IgG (**A**), IgM (**B**), and IgA (**C**) in healthy subjects (HS) and Alzheimer's disease patients (AD) (n = 30 per group). For each antibody isotype, total αGal–HSA-reactive antibodies were measured before ganglioside pre-incubation and compared with residual αGal–HSA binding after pre-incubation with soluble GM1, GM2, or GD1b. Box colors indicate the experimental condition: gray boxes represent total αGal–HSA-reactive antibodies measured before ganglioside pre-incubation; green boxes represent residual αGal–HSA binding after GM1 pre-incubation; blue boxes represent residual αGal–HSA binding after GM2 pre-incubation; and orange boxes represent residual αGal–HSA binding after GD1b pre-incubation. For each color, lighter boxes indicate HS and darker boxes indicate AD. Box plots show the median, 25th and 75th percentiles, and minimum–maximum values; the cross inside each box indicates the mean. Asterisks indicate statistically significant differences ($p < 0.05$). Black asterisks indicate significant differences between HS and AD before ganglioside pre-incubation. Red asterisks indicate significant differences between total αGal–HSA-reactive antibodies and residual binding after ganglioside pre-incubation. Statistical significance was assessed after evaluation of data normality using the Shapiro–Wilk test. Comparisons between HS and AD subjects were performed using an unpaired *t*-test or Mann–Whitney U test, as appropriate. Within-subject comparisons before and after ganglioside pre-incubation were performed using a paired t-test or Wilcoxon signed-rank test, according to the distribution of the paired differences.
